# Rifampicin Mono-Resistance in *Mycobacterium tuberculosis* in KwaZulu-Natal, South Africa: A Significant Phenomenon in a High Prevalence TB-HIV Region

**DOI:** 10.1371/journal.pone.0077712

**Published:** 2013-11-06

**Authors:** Yacoob Mahomed Coovadia, Sharana Mahomed, Melendhran Pillay, Lise Werner, Koleka Mlisana

**Affiliations:** 1 National Health Laboratory Services, University of KwaZulu-Natal, Durban, South Africa; 2 Medical Microbiology Department, University of KwaZulu-Natal, Durban, South Africa; 3 CAPRISA, UKZN, Durban, South Africa; University of Cape Town, South Africa

## Abstract

**Setting:**

The dual epidemics of HIV-TB including MDR-TB are major contributors to high morbidity and mortality rates in South Africa. Rifampicin (RIF) resistance is regarded as a proxy for MDR-TB. Currently available molecular assays have the advantage of rapidly detecting resistant strains of MTB, but the GeneXpert does not detect isoniazid (INH) resistance and the GenoTypeMTBDRplus(LPA) assay may underestimate resistance to INH. Increasing proportions of rifampicin mono-resistance resistance (RMR) have recently been reported from South Africa and other countries.

**Objective:**

This laboratory based study was conducted at NHLS TB Laboratory, Durban, which is the reference laboratory for culture and susceptibility testing in KwaZulu-Natal. We retrospectively determined, for the period 2007 to 2009, the proportion of RMR amongst *Mycobacterium tuberculosis* (MTB) isolates, that were tested for both RIF and INH, using the gold standard of culture based phenotypic drug susceptibility testing (DST). Gender and age were also analysed to identify possible risk factors for RMR.

**Design:**

MTB culture positive sputum samples from 16,748 patients were analysed for susceptibility to RIF and INH during the period 2007 to 2009. RMR was defined as MTB resistant to RIF and susceptible to INH. For the purposes of this study, only the first specimen from each patient was included in the analysis.

**Results:**

RMR was observed throughout the study period. The proportion of RMR varied from a low of 7.3% to a high of 10.0% [overall 8.8%]. Overall, males had a 42% increased odds of being RMR as compared to females. In comparison to the 50 plus age group, RMR was 37% more likely to occur in the 25–29 year age category.

**Conclusion:**

We report higher proportions of RMR ranging from 7.3% to 10% [overall 8.8%] than previously reported in the literature. To avoid misclassification of RMR, detected by the GeneXpert, as MDR-TB, culture based phenotypic DST must be performed on a second specimen, as recommended by the SA NDOH TB guidelines as well as WHO. We suggest that two sputum samples should be obtained at the first visit. The second sputum sample should be stored at 4°C. The latter sample is then readily available for performing additional DST (phenotypic or genotypic) for 2nd lines drugs, resulting in a decreased waiting period for DST results to become available.

## Introduction

The dual epidemics of HIV and TB including MDR-TB are major contributors to high morbidity and mortality rates in South Africa [1], [2] KwaZulu-Natal, South Africa has one of the highest rates of HIV and active Tuberculosis (TB) cases in the world [2], [3].

MDR-TB is defined as resistance to both isoniazid (INH) and rifampicin (RIF), with or without resistance to other anti-tuberculosis drugs [4]. Unlike most other bacteria, resistance in *Mycobacterium tuberculosis*(MTB) develops primarily through mutations in chromosomal genes. These mutations develop spontaneously and are sustained in the bacterial population mainly through selective pressure with inappropriate treatment [5], [6]. Resistant mutants arise at a frequency of 1 in 10^8^ to RIF and 1 in 10^6^ to INH.

Resistance to RIF is largely attributed to nucleotide substitutions in an 81-bp core region of the *rpo*B gene [5]–[7]. In contrast, resistance to INH occurs by mutations in several genes, in particular *inh*A and *kat*G, and to a lesser extent in *ahp*C, *oxy*R, *kas*A, *fur*A and *ndh* [6]–[8]. Separate mutations are required for drug-susceptible strains to become multidrug-resistant ie MDR-TB, as mutations to INH and RIF are not directly linked [7], [9], [10].

RIF resistance is regarded as a proxy for MDR-TB as a large proportion of RIF resistant strains have INH resistance as well [10]–[14]. Theoretically, if RIF resistance and MDR-TB were perfectly correlated, then detection of MDR-TB would be sufficient with a single rapid test that detects RIF resistance. In areas with low RMR but high MDR prevalence, this correlation is particularly applicable. In countries with reports of increasing rates of RMR, this correlation may be questionable and not always applicable [15]–[17].

For the optimal and accurate management of MDR-TB, both a positive culture of MTB and culture based phenotypic drug susceptibility testing (DST) is required. However, conventional methods of culture based phenotypic drug susceptibility testing (DST) are labour intensive and do not provide rapid results [9], [10]. The growing demand for rapid diagnosis has resulted in molecular techniques being increasingly utilized, not only for the detection of MTB, but also MDR-TB [9]. Molecular based tests, such as GenoTypeMTBDR*plus* assay (LPA) (Hain-Lifescience, Germany), which tests for both RIF and INH susceptibility, and the more recently introduced, GeneXpert MTB/RIF assay (Cepheid, USA), which tests for RIF susceptibility only, are being increasingly used in developed countries as well asSouth Africa, for the rapid detection of resistant MTB [9], [10].

Although, molecular assays have the advantage of rapidly detecting resistant strains of MTB, the GeneXpert detects only RIF resistance, whereas the LPA assay detects resistance to both RIF and INH, but underestimates resistance to INH [18]. This underestimation occurs because the LPA detects only a limited number of INH resistant genes. According to published data, the accuracy estimates for INH are variable and the sensitivity is highly heterogeneous across studies, ranging from 57% to 100% [19].

Due to the limitations of molecular based diagnostics, the misreporting of RMR as MDR-TB is probable. This may not only result in falsely elevated rates of MDR-TB being reported, impacting on surveillance data in particular countries, but may also impact on optimal management of patients as well. In an era of total drug resistance and a need for novel anti tuberculosis drugs, any incorrect use of a particular drug will result in increasing resistance and therefore it is important to preserve the drugs we already have.

This study was undertaken in KwaZulu-Natal, South Africa, which has one of the highest rates of HIV and Tuberculosis (TB) co-infections in the world. We retrospectively determined the prevalence of true RIF mono-resistance, using the gold standard of culture based phenotypic drug susceptibility testing (DST), due to limitations of the currently available molecular assays.

## Materials and Methods

### Ethical Considerations

Ethics approval was obtained from the University of KwaZulu-Natal Biomedical Research Ethics Committee (BREC).

### Study Setting

The study was conducted at the National Health Laboratory Services [NHLS], Provincial TB Reference Laboratory, based at Inkosi Albert Luthuli Central Hospital (IALCH). All specimens received were routinely cultured, and if positive on culture, underwent phenotypic drug susceptibility testing during the period 2007–2009. This laboratory currently performs all culture and culture based phenotypic drug susceptibility testing (DST)for the province of KwaZulu-Natal. The laboratory participates in both international and national external, as well as internal quality assurance programs.

### Study Samples

Sputum samples were initially decontaminated with an equal volume of N-acetyl-L-cysteine-sodium hydroxide (NALC-NaOH) and processed using standard laboratory methods. After refrigerated centrifugation, the sediment was cultured on mycobacterial growth indicator tubes (MGIT) with PANTA and OADC (Oleic Acid Dextrose Catalase) and incubated in the BACTEC MGIT 960 system (BD Diagnostics Systems, Sparks, MD). Middlebrook 7H11 agar plates were also inoculated and incubated at 37°C for six weeks. Positive cultures were confirmed as MTB complex using niacin and nitrate and/or Rapid MPT64 antigen assay (Standard Diagnostics, Korea). Positive and negative controls were included in all procedures and tests performed.

### Culture- based phenotypic DST

Culture- based phenotypic DST against both first and second line anti-tuberculosis drugs, using the 1% agar proportion method on Middlebrook 7H10 agar plates, was routinely performed on all specimens received for MCS that were culture positive for MTBC. Standard drug concentrations were used: 1 ug/ml rifampicin, 0.2 ug/ml isoniazid, 7.5 ug/ml ethambutol, 2 ug/ml streptomycin, 5 ug/ml kanamycin and 2 ug/ml ofloxacin. After a 3 week incubation period at 37°C, the drug susceptibility plates were interpreted using a dissecting microscope. For the purposes of this study, only susceptibility to RIF and INH were analysed.

### Statistical Analysis

A Chi-square test for trend was used to determine whether there was a significant trend in RIF mono-resistance over time, categorized by quarter. A logistic regression model was fitted to RMR to determine whether there was an overall difference between genders and age groups. The overall model testing the association between age and RMR adjusted for gender and time (quarter), while the gender-stratified model adjusted for time (quarter).

## Results

Sixty eight thousand seven hundred and twenty six culture positive sputum samples for the period 2007 to 2009 were analysed for RIF susceptibility. Of these, 32.5% were found to be RIF resistant (22,352 of 68,726 samples tested over this period). RMR proportions were analysed within this subpopulation, and are reported in quarterly time periods in the text, figures and tables. Of these RIF resistant cultures, 21 had missing INH susceptibility data and were excluded from the analysis, leaving a total of 22,331 RIF resistant cultures from 16,748 patients. For the purposes of this study, only the first specimen from each patient was included in the analysis.

Of the 16,748 patients data analysed, 431 had missing gender data and 1504 patients had missing age data. The overall median age (IQR) as well as the median age in patients with RMR was 32 (26–40) years respectively. The overall gender distribution was 52.3% females and in patients with RMR, it was 44.9%. Overall, RMR was detected in 8.8% (1,466) of patients during the study period, with a range of 7.3% to 10%. There was no significant trend in RMR over the quarters (2007 to 2009) (p = 0.5240), and no significant trend was observed in either the male or female population (p = 0.4773 and p = 0.5658, respectively); however, overall males had a 42% increased odds of being RMR as compared to females, after adjusting for age and quarter (Odds ratio (OR) 1.42; 93% CI 1.27–1.60; p<0.0001). Table 1 depicts percentages of RMR measured over quarters between 2007 and 2009.

**Table 1:** Rifampicin mono-resistance over quarters from 2007 to 2009 overall and by gender.

% RIF mono-resistance [95% confidence interval] (Number RIF mono-resistant/Total RIF resistant)				
Year	Quarter	Overall*	Females	Males
**2007**	**Q1**	7.3% [5.7%–9.0%] (72/982)	7.5% [5.1%–9.9%] (36/480)	7.1% [4.7%–9.4%] (32/452)
	**Q2**	7.5% [6.1%–9.0%] (99/1312)	5.9% [4.1%–7.8%] (38/639)	8.7% [6.5%–11.0%] (54/618)
	**Q3**	9.3% [7.8%–10.8%] (141/1514)	7.4% [5.5%–9.3%] (53/715)	10.8% [8.5%–13.0%] (79/732)
	**Q4**	10.0% [8.4%–11.6%] (142/1419)	7.9% [5.9%–9.9%] (56/706)	12.6% [10.1%–15.1%] (84/668)
**2008**	**Q1**	8.1% [6.7%–9.4%] (131/1625)	7.4% [5.5%–9.2%] (57/771)	8.9% [6.9%–10.9%] (69/779)
	**Q2**	9.6% [8.1%–11.1%] (140/1465)	8.2% [6.3%–10.1%] (64/780)	10.3% [7.9%–12.6%] (65/633)
	**Q3**	9.2% [7.7%–10.6%] (133/1452)	5.8% [4.1%–7.5%] (42/728)	12.3% [9.9%–14.8%] (84/682)
	**Q4**	8.8% [7.2%–10.4%] (103/1168)	8.3% [6.0%–10.5%] (48/580)	9.0% [6.6%–11.4%] (49/543)
**2009**	**Q1**	8.8% [7.3%–10.2%] (122/1393)	8.3% [6.3%–10.3%] (61/731)	9.2% [7.0%–11.4%] (61/662)
	**Q2**	7.8% [6.4%–9.1%] (117/1502)	7.3% [5.5%–9.1%] (60/822)	8.4% [6.3%–10.5%] (57/680)
	**Q3**	10.0% [8.5%–11.6%] (147/1463)	8.8% [6.8%–10.7%] (69/787)	11.5% [9.1%–13.9%] (78/676)
	**Q4**	8.2% [6.8%–9.6%] (119/1453)	6.4% [4.7%–8.1%] (51/794)	10.3% [8.0%–12.6%] (68/659)
**Total**		**8.8% [8.3%–9.2%]**	**7.4%[6.9%–8.0%]**	**10.0% [9.4%–10.7%]**
		**(1466/16748)**	**(635/8533)**	**(780/7784)**

* A total of 431 patients had missing gender data.

We grouped ages in the following categories: <18, 18–24, 25–29, 30–39, 40–49 and 50+ years and assessed RMR over time. Those aged 25 to 29 years had a significant increase in RMR over time (p = 0.0094), in comparison to the 50 plus age group, while those aged 30 to 39 years had a moderate association for a decreasing trend over time (p = 0.0554). No trends were observed in the other age groups.

As shown in Table 2, 25 to 29 year olds were at 37% greater odds of having RMR, in comparison to 50+ year olds (OR 1.37; 95% CI 1.08–1.73; p = 0.0102). However, in females there appears to be no significant difference between the age groups and the significant difference between 25 to 29 and 50+ year olds is largely driven by the male population. Males aged 25 to 29 years are at 64% increased odds of having RMR compared to 50+ year olds (OR 1.64; 95% CI 1.21–2.23; p = 0.0016). Although not statistically significant, 30 to 39 year olds were at a 27% greater odds of having RMR compared to 50+ year olds (p = 0.0867.

**Table 2:** Adjusted logistic regression of being Rifampicin mono-resistant, assessing the effect of age, overall and stratified by gender.

Age group	Overall*		Females**		Males**	
(years)	Odds Ratio (95% CI)	p-value	Odds Ratio (95% CI)	p-value	Odds Ratio(95% CI)	p-value
<18	0.99 (0.72–1.35)	0.9316	0.97 (0.60–1.55)	0.8831	0.98 (0.63–1.51)	0.9156
18–24	1.04 (0.80–1.34)	0.7699	1.14 (0.78–1.68)	0.5005	0.84 (0.58–1.21)	0.3482
25–29	1.37 (1.08–1.73)	0.0102	1.12 (0.77–1.63)	0.5693	1.64 (1.21–2.24)	0.0016
30–39	1.17 (0.94–1.46)	0.1562	1.03 (0.72–1.48)	0.8759	1.27 (0.97–1.68)	0.0867
40–49	1.08 (0.85–1.38)	0.5103	1.18 (0.79–1.75)	0.4181	1.03 (0.76–1.40)	0.8561
50+	1.00(ref)	–	1.00(ref)	–	1.00 (ref)	–

* Adjusting for gender and quarter.

** Adjusting for quarter.

## Discussion

RMR was observed throughout the study period of 2007 to 2009. The proportion of RMR varied from a low of 7.3% to a high of 10.0%, with an overall proportion of 8.8%. To the best of our knowledge there is a scarcity of data on resistant TB, and in particular, RMR from African countries. However, in South Africa, two studies have shown that RIF mono resistance is not infrequent.

A study from Cape Town, South Africa by Mukinda *et al* showed significantly increasing trends in RMR over a 5 year period (2004–2008). During this period RMR cases more than tripled, from 31 cases in 2004 to 98 cases in 2008 [15]. Dramowski *et al* also reported that RMR disease is increasingly encountered, particularly among HIV-infected and HIV-exposed non-infected children in the Cape province [16].

Similarly, other parts of the world have also reported that RMR is not uncommon. Sanders *et al* reported that levels of drug resistance in Bujumbura are higher than average for Africa and most worrying was the appearance of MDR-TB and RMR in new cases [17]. Traore *et al* studied a large number of MTB complex clinical isolates from diverse countries to detect RMR. He reported a median of 4.6% RMR with the highest prevalence in Western Europe [20]. Pablos-Mendez *et al* performed anti-TB drug resistance surveillance (WHO) in 19 different countries and reported RMRin all. A median of 1.5% was noted, with the highest rate of 6.9% being reported from both Thailand and Dominican Republic [21]. In comparison to the above studies, we report a higher proportion of RMR.

HIV/TB co-infection occurs commonly in our setting with as high as 70% of TB infected individuals co-infected with HIV [2], [22]. RMR has previously been correlated with HIV positivity rates. A survey of TB isolates collected in the United States between 1993 and 1996 documented RMR of 2.6% in HIV-positive cases and only 0.2% in HIV-negative cases [23]. Similarly, Sandman *et al* in New Yorkshowed a RMR prevalence rate of 1,4%, with 81% of cases being detected in HIV positive patients [24]. Although we did not have access to data on HIV positivity, which is a major limitation of this study, it is widely recognised that KZN has one of the highest HIV prevalence rates in the world. Therefore, the higher proportion of RMR observed in our study population is most likely explained by the very high prevalence of HIV.

RMR linked to rifabutin prophylaxis has previously been documented in studies from developed countries [25]. Rifabutin prophylaxis to prevent *Mycobacterium avium* complexis not currently practiced in South Africa. The increase in RMR in our study could possibly be attributed to other factors, such as HIV associated malabsorption of anti-TB drugs, increased rates of extra-pulmonary tuberculosis, previous incorrect management of TB, adherence challenges and antiretroviral and anti-TB drug interactions [26]–[28].

In the current study, males were more likely to have RMR. Incident rates of tuberculosis in South Africa have shown higher prevalence rates in males than in females This gendered incidence was associated with health seeking behaviour and occupation [29]. However, to the best of our knowledge, gender as a specific risk factor for RMR has not previously been documented by others.

Although using age as risk factor is tenous, in this study RMR was more likely to occur in the 25–29 year age group. It is well recognised that HIV is more common in this age group and that RMR is more common in HIV as previously highlighted [26]–[28]. Our findings however are in contrast to Mukinda *et al* who reported RMR higher in >40 year age group [15].

It is important to note that we did not specifically address the reliability of RIF resistance detected by molecular tests as a proxy for MDR-TB. Nevertheless, our findings, using culture –based phenotypic DST as the gold standard, indicate a significant proportion of RMR ranging from 7.3% to 10% (overall 8.8%) in KZN, South Africa. If molecular assays are to be more widely used, it is essential that confirmatory culture based phenotypic DST, as recommended by WHO, be performed, particularly in geographical areas with high RMR rates. The South African National Department of Health TB guidelines also clearly state that a second sputum specimen must be sent in cases of RIF resistance detected by GeneXpert. This approach circumvents the misclassification of RMR as MDR-TB, which is particularly relevant in countries where RIF resistance, detected by the GeneXpert, is initially recorded as MDRTB in TB registers.

Regarding the LPA assay, the converse is true, in that it underestimates INH resistance, and may be erroneously reported as RMR. The accuracy estimates for INH are variable and the sensitivity is heterogeneous across studies, ranging from 57% to 100% [19]. In a study carried out in Uganda, the sensitivity, specificity, positive and negative predictive values were: 80.8%, 100.0%, 100.0% and 93.0% respectively for detection of isoniazid resistance [30]. A sensitivity, specificity, positive and negative predictive value of 94.2, 99.7, 99.1, and 97.9% was reported from a study carried out in South Africa [9].

Currently, culture based phenotypic DST results become available in 21 days or more under ideal conditions. Results may further be delayed due to contaminated laboratory cultures, delays in tracking patients and transfer of patients to different health facilities. Attending clinicians may therefore not obtain culture- based phenotypic DST results timeously, and many cases of RMR will continue to be erroneously classified as MDR-TB. Furthermore, therapy for RMR-TB has not yet been optimised, with many cases currently being treated with potentially toxic and expensive regimens used for MDR-TB [31]. The SA NDOH recommends that these cases be treated as MDR-TB with the addition of INH for 18 months [32].

We report a higher proportion of RMR, ranging from 7.3% to 10%, (overall 8.8)] than previously reported in the literature. To avoid misclassification of RMR, detected by the GeneXpert, as MDR-TB, culture based phenotypic DST must be performed on a second specimen, as recommended by the SA NDOH TB guidelines as well as WHO.

In conclusion, we suggest that two sputum samples should ideally be obtained at the first visit The second sputum sample should be stored at 4°C. The latter sample is then readily available forperforming additional DST (phenotypic or genotypic) for 2nd line drugs. This will therefore decrease the waiting period for DST results to become available.
